# Magic Numbers and Mixing Degree in Many-Fermion Systems

**DOI:** 10.3390/e25081206

**Published:** 2023-08-14

**Authors:** D. Monteoliva, A. Plastino, A. R. Plastino

**Affiliations:** 1 UNLP-Comisión de Investigaciones Científicas Provincia de Buenos Aires La Plata, La Plata 1900, Argentina; monteoli@fisica.unlp.edu.ar; 2 Instituto de Física La Plata—CCT-CONICET, Universidad Nacional de La Plata, La Plata 1900, Argentina; 3 CeBio-Departamento de Ciencias Básicas, Universidad Nacional del Noroeste, Prov. de Buenos Aires (UNNOBA), CONICET, Junin 6000, Argentina; arplastino@unnoba.edu.ar

**Keywords:** Tsallis entropy, many-fermion systems, mixture degree, finite temperature, magic numbers

## Abstract

We consider an *N* fermion system at low temperature *T* in which we encounter special particle number values $N_{m}$ exhibiting special traits. These values arise when focusing attention upon the degree of mixture (DM) of the pertinent quantum states. Given the coupling constant of the Hamiltonian, the DMs stay constant for all *N*-values but experience sudden jumps at the $N_{m}$. For a quantum state described by the matrix ρ, its purity is expressed by $Tr\rho^{2}$ and then the degree of mixture is given by $1-Tr\rho^{2}$, a quantity that coincides with the entropy $S_{q}$ for q=2. Thus, Tsallis entropy of index two faithfully represents the degree of mixing of a state, that is, it measures the extent to which the state departs from maximal purity. Macroscopic manifestations of the degree of mixing can be observed through various physical quantities. Our present study is closely related to properties of many-fermion systems that are usually manipulated at zero temperature. Here, we wish to study the subject at finite temperature. The Gibbs ensemble is appealed to. Some interesting insights are thereby gained.

## 1. Introduction

Tsallis q-entropy, also known as non-extensive entropy, is an alternative entropy measure introduced by Constantino Tsallis in 1988. Unlike the traditional Shannon entropy or Boltzmann–Gibbs entropy, which are based on logarithmic functions, Tsallis entropy incorporates a power-law function to capture certain characteristics of diverse physical scenarios, in particular, those involving complex systems. For instance, Tsallis entropy has been used to describe physical systems that exhibit long-range interactions, such as self-gravitating systems, turbulent flows, and systems with power-law distributions [1,2,3,4,5,6,7,8,9,10,11,12,13,14]. It provides a framework to characterize the statistical properties of these systems and has connections to generalized statistical mechanics and information theory. It is worth noting that Tsallis entropy has its own set of mathematical properties and implications, and its interpretation and applicability depend on the context and field of study [1,2,3,4,5,6,7].

Tsallis entropy has also been used to investigate a various range of quantum phenomena (see, for example, [8,9,10,11,12,13,14] and references therein). Some of these studies deal with the explicit application of Tsallis thermostatistics to describe particular quantum systems. It is worth noting, however, that Tsallis entropy also proved to be valuable for the analysis of quantum phenomena not related to Tsallis thermostatistics. In this sense, Tsallis entropy is already an important member of the general tool-kit employed by quantum scientists. Indeed, *Tsallis entropy* can nowadays be found mentioned in monographs devoted to aspects of quantum science, such as quantum entanglement [15] or quantum information [16], which are not necessarily linked to the Tsallis statistical theory. In particular, the entropy $S_{q}$, associated with the value q=2 of the Tsallis parameter, which is sometimes referred to as *linear entropy*, is a widely used measure of the degree of mixedness exhibited by a quantum state.

### Present Goal

The aim of the present effort is to employ the $S_{2}$ entropy to characterize some features of many-fermion systems at low temperature, which constitute finite-temperature remnants of basic properties, related to quantum phase transitions, exhibited by these systems at zero temperature.

In particular, this work is devoted to studying properties of the quantum mixing-degree quantifier and of its manifestations at finite, but very low, temperatures.

## 2. Preliminaries

### 2.1. Quantum Mixing-Degree Quantifier

In quantum mechanics, quantum states can exist in two fundamental forms: pure states and mixed states. A pure state is a state that can be described by a single, normalized wave function, and it exhibits maximal coherence and well-defined quantum properties. On the other hand, a mixed state is a statistical ensemble of pure states, each with its associated probability. It exhibits less coherence and may have probabilistic uncertainties. The degree of mixing or superposition in a quantum state is measured here by the mixing quantifier $C_{f}$.

$C_{f}$ is equal to unity less than the quantum purity $P_{y}$. The purity of a quantum state quantifies its coherence and is a measure of how close the state is to being pure. It is defined as the trace of the square of the state’s density matrix ρ as $P_{y}=Tr\left(\rho^{2}\right)$. For a pure state, the purity is equal to 1, while for a mixed state, the purity is less than 1.

### 2.2. Usefulness of Exactly Solvable Many-Body Systems

In this work, we employ an exactly solvable model. Exactly solvable many-body systems are of great importance and usefulness in various areas of physics and related disciplines. These systems are analytically solvable, meaning their quantum states, dynamics, and properties can be described using closed-form mathematical expressions. Their usefulness stems from the deep insights they provide into the behavior of complex quantum systems, as well as their role in serving as benchmarks for testing and developing theoretical methods. Here are some key advantages and applications of exactly solvable many-body systems:

- Insight into quantum phenomena: Exactly solvable many-body systems often serve as simple and tractable models that exhibit essential quantum phenomena, such as quantum phase transitions, entanglement, and quantum correlations. They provide valuable intuition and understanding of fundamental quantum concepts.
- Testing quantum theories: Because these systems are analytically solvable, they are ideal for testing and validating theoretical methods and approximations used in more complicated systems. They allow researchers to check the accuracy and efficiency of numerical algorithms and analytical techniques.
- Educational tools: Exactly solvable many-body systems are commonly used as educational tools in teaching quantum mechanics and statistical physics. They provide students with concrete examples to illustrate abstract concepts and principles.
- Foundation for approximations: Many-body systems that are exactly solvable often serve as the foundation for developing approximate methods applicable to more complex systems. These methods include mean-field theory, perturbation theory, and variational approaches.
- Condensed matter physics: Exactly solvable models play a crucial role in understanding phase transitions and critical phenomena in condensed matter physics. They shed light on the emergence of collective behaviors in large systems.
- Quantum information theory: Solvable models are essential in quantum information theory, particularly in studies related to quantum computing, quantum error correction, and quantum communication protocols.
- Benchmarking numerical techniques: Exactly solvable models provide precise results that can be used as benchmarks to assess the accuracy and efficiency of numerical techniques, such as Monte Carlo simulations, tensor network methods, and a density-matrix renormalization group (DMRG).

In summary, exactly solvable many-body systems are indispensable tools in understanding and exploring quantum phenomena, testing theoretical methods, and providing insights into the behavior of complex quantum systems. Their importance extends beyond theoretical physics and has applications in condensed matter physics, quantum information, and related fields. In nuclear physics, a model of this type that has enjoyed considerable attention is the so-called Lipkin one [17,18]. We discuss here a variant of such a model.

### 2.3. Using Very Low Temperature Statistical Mechanics Techniques to Approximate Ground-State Properties

Using very low temperature statistical mechanics techniques is a powerful and common approach to approximate ground-state properties of quantum systems. Ground-state properties are of fundamental importance as they represent the system’s lowest energy state, and understanding them is crucial for gaining insights into the system’s behavior and properties. At very low temperatures (close to absolute zero), thermal fluctuations become negligible, and the system tends to occupy its ground state more predominantly. This allows for various low-temperature approximations that simplify the analysis and computation of ground-state properties.

This procedure, **which we use in this work**, is an essential tool for studying ground-state properties in various physical systems, including condensed matter physics, quantum chemistry, and quantum information theory. They allow researchers to gain insights into the behavior of complex quantum systems and provide a foundation for understanding and engineering quantum materials and technologies. Concomitant references are given below.

### 2.4. Magic Numbers in Many-Fermion Systems

In the context of nuclear physics, “magic numbers” refer to specific numbers of protons or neutrons in atomic nuclei that correspond to particularly stable and strongly bound configurations. These magic numbers are associated with closed-shell configurations, which have special quantum properties resulting in enhanced stability and distinct nuclear properties. For protons, the magic numbers are 2, 8, 20, 28, 50, 82, and 126, representing the number of protons needed to fill complete shells in the nuclear potential. For example, the nuclei with proton numbers 2, 8, 20, 28, 50, 82, and 126 (helium-4, oxygen-16, calcium-40, nickel-48, tin-100, and lead-208, respectively) are particularly stable and are known as “doubly magic” nuclei. Similarly, for neutrons, the magic numbers are 2, 8, 20, 28, 50, 82, and 126, representing the number of neutrons needed to fill complete shells in the nuclear potential. Nuclei with both proton and neutron magic numbers are especially stable and have unique nuclear properties.

Magic numbers play a crucial role in the nuclear structure and have significant implications in various nuclear processes, such as nuclear reactions and nuclear astrophysics. They also form the basis for understanding the behavior of nucleons (protons and neutrons) in the nuclear potential and are essential for interpreting nuclear data and predicting nuclear properties.

The concept of magic numbers extends beyond nuclear physics to other many-fermion systems, such as atomic and molecular clusters, where similar patterns of enhanced stability due to closed-shell configurations can be observed. Magic numbers in these systems have important consequences for their chemical and physical properties. Overall, magic numbers are fundamental in understanding the structure and stability of many-fermion systems and have far-reaching implications in various areas of physics and chemistry.

We will find them here, in an abstract many-fermions system.

### 2.5. Expanding on Our Present Objectives

The quantum *N*-fermion system exhibits various properties, some of them indeed intricate [10,19,20,21,22,23,24,25,26,27,28,29,30,31]. We will study manifestations of quantum properties at a very low finite temperature. How? As described by statistical mechanics and with reference to an exactly solvable model. This model is able to illuminate some interesting theoretical effects. We speak of a many-fermion model of the Hubbard model kind [28].

As stated above, thermal statistical manipulation of many-fermion body behavior at finite temperature can yield interesting insights [29]. Accordingly, we appeal here to an exactly solvable Lipkin-like model (LLM) [17,18] at finite temperature and consider the pertinent structural traits in the framework of Gibbs’ canonical ensemble formalism. LLMs are nontrivial, finite, easily solvable fermion systems [17,18]. Indeed, they are quite useful testing grounds for envisaging new many-body approaches and using them, as we always have, for an exact solution with which to compare our approximations. In this effort, we work with one of the Lipkin model variants, called the AFP (Abecasis–Faessler–Plastino) model [26,32,33,34].

## 3. The AFP Model Structure

The AFP model can be regarded as a very simplified atomic nucleus containing *N* nucleons in just two levels. It is exactly solvable. The model considers a quite simple fermion–fermion interaction of strength *v*. In nature, of course, the coupling constants are fixed. In the model, of course, we vary it so as to observe how much the ground-state traits are affected by *v* changes. We also study the model behavior for different *N*, as we have in nature nuclei with quite distinct nucleon numbers, whose ground states display quite different traits.

Our model possesses N=2Ω fermions that occupy two different N-fold degenerate single-particle (sp) energy levels. They are characterized by an sp energy gap ϵ. This entails 4Ω sp microstates. Two quantum numbers (μ=±1 and p=1,2,…,N) are associated with a given microstate p,μ>. The first one, called μ, adopts the values μ=−1 (lower level) and μ=+1 (upper level). The second runs from unity to *N*. This remaining quantum number, called *p*, is baptized as a quasi-spin or pseudo-spin, which singles out a specific microstate pertaining to the 2N-fold degeneracy. In the pair p, μ is viewed as a “site” that can be occupied (by a fermion) or empty. Lipkin fixes

(1) N=2J.

Here, *J* is a sort of angular momentum. Lipkin [17,29] uses special operators called quasi-spin ones. Below, we use the usual creation operators $C_{p,\mu }^{+}$ and the associated destruction ones $C_{p,\mu }$ for creating or destroying a fermion at a site |p,μ>.

### 3.1. Quasi-Spin Operators

Quasi-spin operators *J* are mathematical constructs used to describe certain collective properties of a many-body system. These operators arise in various areas of physics, such as nuclear physics, condensed matter physics, and quantum optics, where systems can exhibit collective behavior due to interactions between constituent particles. Quasi-spin operators are particularly useful in cases where the collective behavior resembles the behavior of spin systems, hence the name “quasi-spin”. The concept of quasi-spin originates from the analogy between the properties of many-body systems and those of spin systems, which are well-understood and widely used in quantum mechanics. In a spin system, the angular momentum operators (spin operators) obey the commutation relations of the SU2 algebra, and they play a fundamental role in characterizing the system’s angular momentum and magnetic properties. In many-body systems, the quasi-spin operators are introduced to represent collective excitations or modes that behave similarly to angular momentum. These operators often have algebraic properties resembling the SU2 algebra, making them suitable for describing the collective dynamics of the system. Overall, quasi-spin operators offer a valuable tool in theoretical physics for investigating collective behavior in complex many-body systems, facilitating the understanding of emergent phenomena, and enabling the development of analytical and numerical techniques to study these systems in different physical contexts.

In nuclear physics, for example, in the AFP model considered here, one utilizes quasi-spin operators to describe the collective behavior of nucleons in a nucleus. The specific form and properties of the quasi-spin operators depend on the nature of the many-body system being studied and the interactions between its constituents. They are introduced to simplify the description of collective phenomena and, as stated above, provide a powerful mathematical framework for treating many interacting fermions. One has for these operators the definitions

(2) $$J_{z}=\sum_{p,\mu }\mu \;C_{p,\mu }^{+}C_{p,\mu },$$

(3) $$J_{+}=\sum_{p}C_{p,+}^{+}C_{p,-},$$

(4) $$J_{-}=\sum_{p}C_{p,-}^{+}C_{p,+},$$

and the Casimir operator

(5) $$J^{2}=J_{z}^{2}+\frac{1}{2}\left(J_{+}J_{-}+J_{-}J_{+}\right).$$

The eigenvalues of $J^{2}$ take the form J(J+1) and the Lipkin Hamiltonian reads (*v* is a coupling constant)

(6) $$H=\epsilon J_{z}+\frac{v}{4}\left(J_{+}^{2}+J_{-}^{2}\right).$$

### 3.2. The AFP Model

It displays [26,32,33,35] a similar quasi-spin structure. One uses the operators

(7) $$G_{ij}=\sum_{p=1}^{2\Omega }C_{pi,}^{+}C_{p,j}$$

Also, *v* is the two-body-interaction coupling constant. Our Hamiltonian is

(8) $$H_{AFP}=\epsilon \sum_{i}^{N}G_{i,i}+V\left(J_{x}-J_{x}^{2}\right).$$

$J_{x}$ is the sum $[J_{+}+J_{-}]/2$. Its eigenvalues are $E_{n}\left(c,J\right)$ [17,18].

For the AFP Hamiltonian matrix, please see Appendix A.

## 4. Working within the Gibbs Ensemble Framework

The procedure is described in detail in [35]. All thermal quantities of interest are deduced from the partition function *Z* [19]. We construct *Z* using probabilities assigned to the models’ microscopic states. Their energies are $E_{i}$ [19]. Some important macroscopic quantifiers are computed as in [19]. These indicators, together with *Z*, derive from the canonical probability distributions [19] $P_{n}\left(v,J,\beta \right)$. β is the inverse temperature. The pertinent expressions are given in [19]. We call the mean energy *U* and the free energy *F*:

(9) $$P_{n}\left(v,J,\beta \right)=\frac{1}{Z(v,J,\beta )}e^{-\beta E_{n}\left(v,J\right)}$$

(10) $$Z\left(v,J,\beta \right)=\sum_{n=0}^{N}e^{-\beta E_{n}\left(v,J\right)}$$

(11) $$\begin{array}{cc} U(v,J,\beta ) & =\langle E\rangle =-\frac{\partial \ln Z(v,J,\beta )}{\partial \beta }\\ \; & =\sum_{n=0}^{N}E_{n}\left(v,J\right)P_{n}\left(v,J,\beta \right)\\ \; & =\frac{1}{Z(v,J,\beta )}\sum_{n=0}^{N}E_{n}\left(v,J\right)e^{-\beta E_{n}\left(v,J\right)} \end{array}$$

(12) $$S\left(v,J,\beta \right)=1-\sum_{n=0}^{N}P_{n}\left(v,J,\beta \right)\ln\left[P_{n}\left(v,J,\beta \right)\right]$$

(13) F(v,J,β)=U(v,J,β)−TS(v,J,β).

The thermal quantifiers above provide much more information than the one obtained via just the quantum resources of zero temperature *T* [19]. Taking a low enough *T*, our quantifiers above yield a good representation of the T=0 scenario [19]. Below, we will adopt the high enough β=20 value.

### A State’s ρ Degree of Mixture $C_{f}$

As is well-known in quantum mechanics, the degree of mixture $C_{f}$ of a given state represented by ρ is given by [36]

(14) $$C_{f}=1-Tr\rho^{2}=1-\sum_{i}p_{i}^{2},$$

where $Tr\rho^{2}$ is the so-called “Purity” $P_{y}$. Note that we have $C_{f}=0$ and $P_{y}=1$ for pure states. $C_{f}$ is a very important quantity for us here. Because the Tsallis practitioner will immediately recognize that Equation (14) is Tsallis’ entropy of index q=2, i.e., $S_{2}$. One encounters a direct link (equality) between $S_{2}$ and $C_{f}$.

In probability terms, one has $P_{y}=\sum_{n=0}^{N}{\left(P_{n}\left(v,J,\beta \right)\right)}^{2}$ and $C_{f}=S_{2}=1-P_{y}^{2}$.

## 5. Present Results for Our Main Quantifier $S_{2}$

### 5.1. Results as a Function of the Particle Number

**Figure 1:**
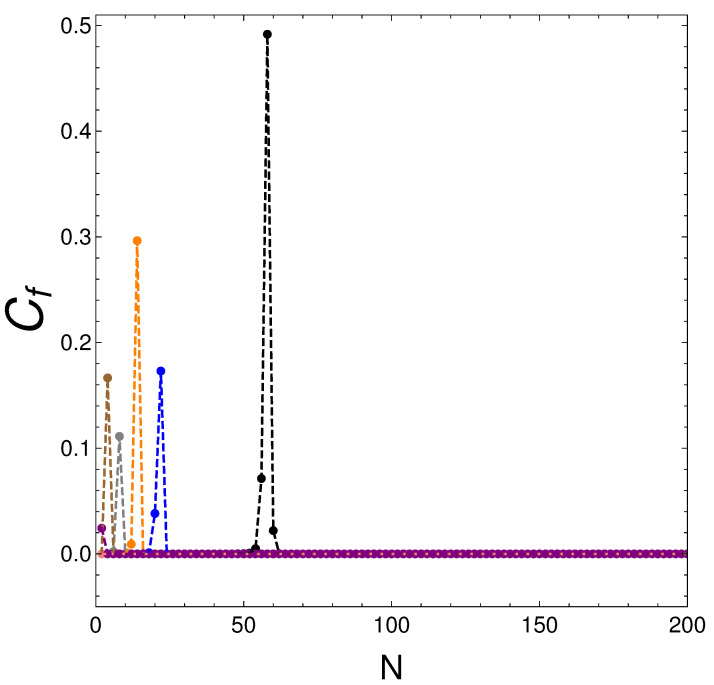
We plot $C_{f}=S_{2}$ vs. *N* for several *v*-values, with β=20. Purity prevails, with intriguing exceptions. v—colors are assigned in this way: v=0.5 (violet); v=0.3 (rose); v=0.2 (brown); v=0.1 (grey); v=0.05 (orange); v=0.03 (blue); v=0.01 (black); v=0.001 (green); v=0 (red).

Remember that we work at finite temperature but for very low *T* values, so that T=0 remnants are very pronounced ones. In our first graph (Figure 1), we depict $S_{2}=C_{f}$ versus the fermion number for several values of the coupling constant *v*. Remarkably enough, given the *v* value, for all *N* values but one, $S_{2}=C_{f}=0$, entailing *finite-temperature purity: T is not high enough to generate mixing*. This is an interesting result. However, given *v*, this happens for specific values of *N* and only for them.

This effect occurs for **all *v*** and we encounter a special *N* value ($=N_{m}$) for which $S_{2}$, and the mixing degree, suddenly grows. Here, we borrow the described “magic number” $N_{m}\left(v\right)$ from nuclear physics such that the system experiences a noticeable amount of mixing. Magic numbers are rather typical features of fermion systems. We discover that as *v* diminishes, $N_{m}$ grows.

**Figure 2:**
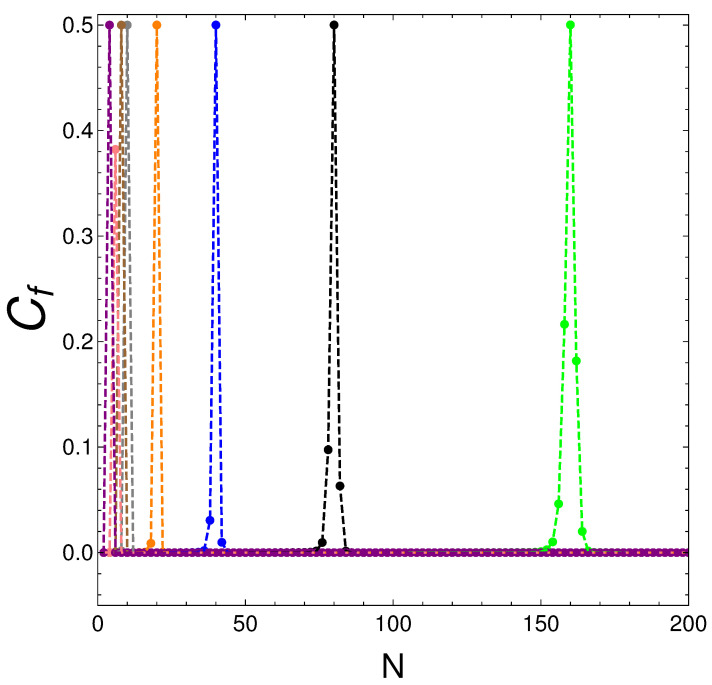
We plot $C_{f}$ versus *N* for the *v*-values listed in Table 1. The peaks occur at the corresponding *N* values of Table 1. However, we see that $C_{f}$ ceases to be zero for some fermion numbers that are neighbors of $N_{m}$, which are marked with dots in the graph.

Let us now discuss the results depicted in Figure 2 below. One notices there that given *N*, $C_{f}$ vs. *v* presents a peak at a particular value of *v*, where $C_{f}=0.5$. We look at these special values in Figure 2:

### 5.2. Energetic Interpretation of the $N_{m}$

Let $E_{0}\left(N\right)$ stand for the energy of the ground state of our Hamiltonian matrix and, further, let $E_{1}\left(N\right)$ be the energy of the associated first excited state. Consider their difference, that is, the excitation energy of the first level above the ground state.

(15) $$A\left(N\right)=E_{1}-E_{0}.$$

We see in Table 1 that these two energies are much closer to each other for $N_{m}$ than for $N_{m-1}$ or $N_{m+1}$. With regard to Figure 1, we next list in Table 1 the energy differences *A* for several number-of-particles triplets, $N_{m-2}$, $N_{m}$, and $N_{m+2}$. These triplets are associated with the peaks in Figure 1, in the way we discuss next.

At $N_{m}$, we see that the energy difference *A* is very small, which in turn generates a sort of quasi-degeneracy of the two lowest-lying states of our Hamiltonian matrix, which favors mixing. *A* is instead larger for $N_{m\pm 1}$ than for $N_{m}$.

### 5.3. Results as a Function of the Coupling Constant v

**Figure 3:**
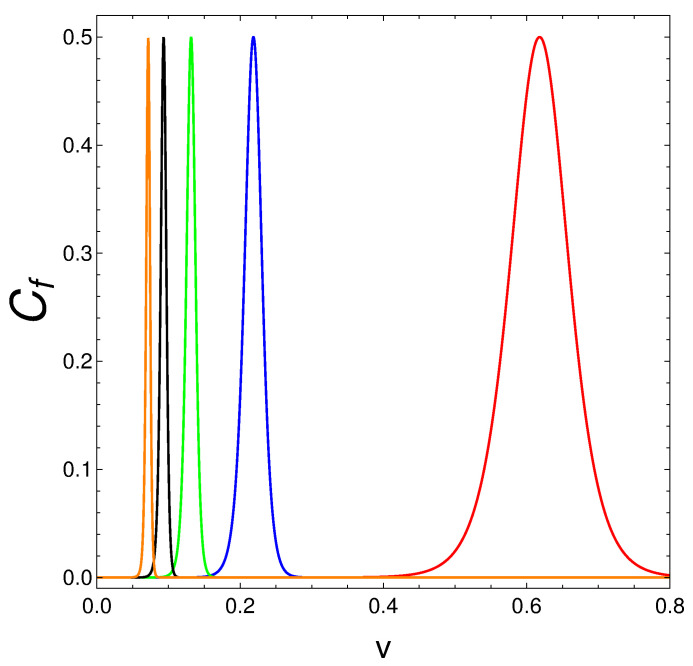
We plot $C_{f}=S_{2}$ vs. *v* for β=20. Colors are as follows: N=2 (red); N=4 (blue); N=6 (green); N=8 (black); N=10 (orange). See that we confront here magic *v*-regions (windows), whose size diminishes as *N* grows. Outside these windows, the mixing degree vanishes.

We now consider the behavior of the mixing degree $C_{f}=S_{2}$ as a function of the Hamiltonian’s coupling constant *v* for different values of *N*. See Figure 3, which displays an illustrative example. Even if purity prevails overall, magic numbers become noticeable again, but this time with reference to *v* values. We have a magic number for every *v*.

### 5.4. Effects of the $S_{2}$ Peaks on Macroscopic Quantities

**Figure 4:**
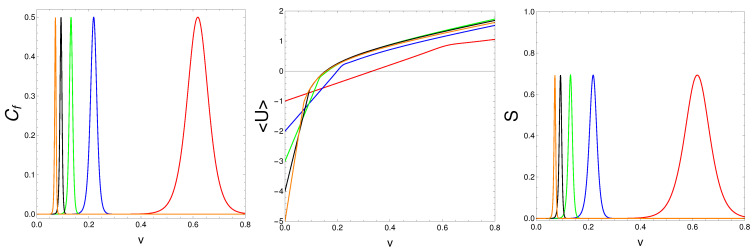
We find $C_{f}=S_{2}$ (**left**), <*U*> (**center**), and Shannon’s *S* (**right**) confront vs. *v* for N=2,4,⋯,10, with β=20. One sees that <*U*> displays slope changes at the *v* values associated with entropic peaks. Regarding the trait <*U*>, this fact shows the existence of critical values for the coupling constants at which the mean energy suffers a slope change. These critical values are found within the areas covered by the $S_{2}$ peaks.

Let us compute the mean energy <*U*>, and the Shannon entropy *S* versus *v*. The results are depicted in Figure 4. The magic character manifests itself in slope changes for the mean energy and in peaks for the two entropies.

## 6. Conclusions

Statistical mechanics often appeals to probability models so as to describe the behavior of systems composed of a large number of microscopic constituents. In this work, our constituents are interacting fermions and the ensembles are the canonical Gibbs ones. We work at very low temperatures so as to use results as useful proxies for many-body features at zero temperature. Remnants of these results survive very well at low *T* and are much easier to deal with than appealing directly to the many-fermions system’s structural properties. We have appealed to a well-known exactly solvable many-fermion system so as to discuss exact results. More specifically, we have investigated fermion dynamical traits associated to the mixing degree of the pertinent many-body states using Tsallis entropy for q=2.

There are two important quantities in this paper: the fermion number *N* (a quantity that in a sense defines the system (think of an atomic nucleus)) and the Hamiltonian’s coupling constant *v*, which is a mere (although very important) parameter. Considering the system’s microstates (MS) at a very low temperature, we find that, given the *v*-value, the MS remain pure, at our finite low *T*, for all *N*, but with the exception of a special one, which we call “magic” and denote by $N_{m}$. Magic numbers are typical in fermion systems [23]. Here, for each *v*, there is a corresponding $N_{m}$, which is smaller the larger the coupling constant is. Table 1 assigns responsibility for the existence of magic numbers to a quasi-degeneracy of the ground state and the first excited one. This happens, of course, at zero temperature, but remnants of such a trait persist at low temperatures. The special quantities, which we call magic, are discrete (of course). One has $C_{f}=0.5$ at the peaks.

We emphasize that the magic mixing degree *is not caused by temperature*. It originates, as stated above, in a quasi-degeneracy of the Hamiltonian’s two lowest-lying eigen-energies.

## Figures and Tables

**Table 1:** Values of the energy difference $A\left(N\right)=E_{1}-E_{0}$ for the number-of-particles triplets associated with the peaks in Figure 1. The values at the center of the triplet exhibit quasi-degeneracy as likely being responsible for the magic number peculiarity. That is, the two energies $E_{1}$ and $E_{2}$ are much closer to each other for $N_{m}$ than for $N_{m-1}$ or $N_{m+1}$.

Color Line	*v*	$N_{m}$	$A_{m-2}$	$A_{m}$	$A_{m+2}$
Black	0.01	58	0.1629	0.0129	0.2243
Blue	0.03	22	0.1959	0.1123	0.5503
Orange	0.05	14	0.2689	0.0755	0.6447

## Data Availability

Every thing needed is found in the manuscript.

## Appendix A. Our Hamiltonian Matrix

For the AFP, one deals with ( see from Equation (6) of [33]) the Hamiltonian matrix:

(A1) $$\begin{array}{cc} \langle n^{\prime }|H_{AFP}|n\rangle = & \left(n-J\right)\delta_{n^{\prime },n}+\frac{1}{2}v\{2\left(2J^{2}+J+n^{2}-2Jn\right)\delta_{n^{\prime },n}\\ \; & +2\sqrt{\left(2J-n)(n+1\right)}\delta_{n^{\prime },n+1}+2\sqrt{(2J-n+1)n}\delta_{n^{\prime },n-1}\\ \; & -\sqrt{\left(2J-n-1)(n+2)(2J-n)(n+1\right)}\delta_{n^{\prime },n+2}\\ \; & -\sqrt{(2J-n+2)(n-1)(2J-n+1)n}\delta_{n^{\prime },n-2} \end{array}$$
